# Supplementary material for: Causal interplay between lactose intolerance and gut microbiota: a combined bidirectional Mendelian randomization and in vivo validation study
Source: Front Nutr. 2026 Jun 1;13:1803337. doi: 10.3389/fnut.2026.1803337 (PMC13265576; doi:10.3389/fnut.2026.1803337)
Supplement: Supplementary file 1 [file Data_Sheet_1.zip › supplementary materials/Forward/leave one out cross validation/ebi-a-GCST90027715.finngen_R12_E4_LACTONAS.pdf]

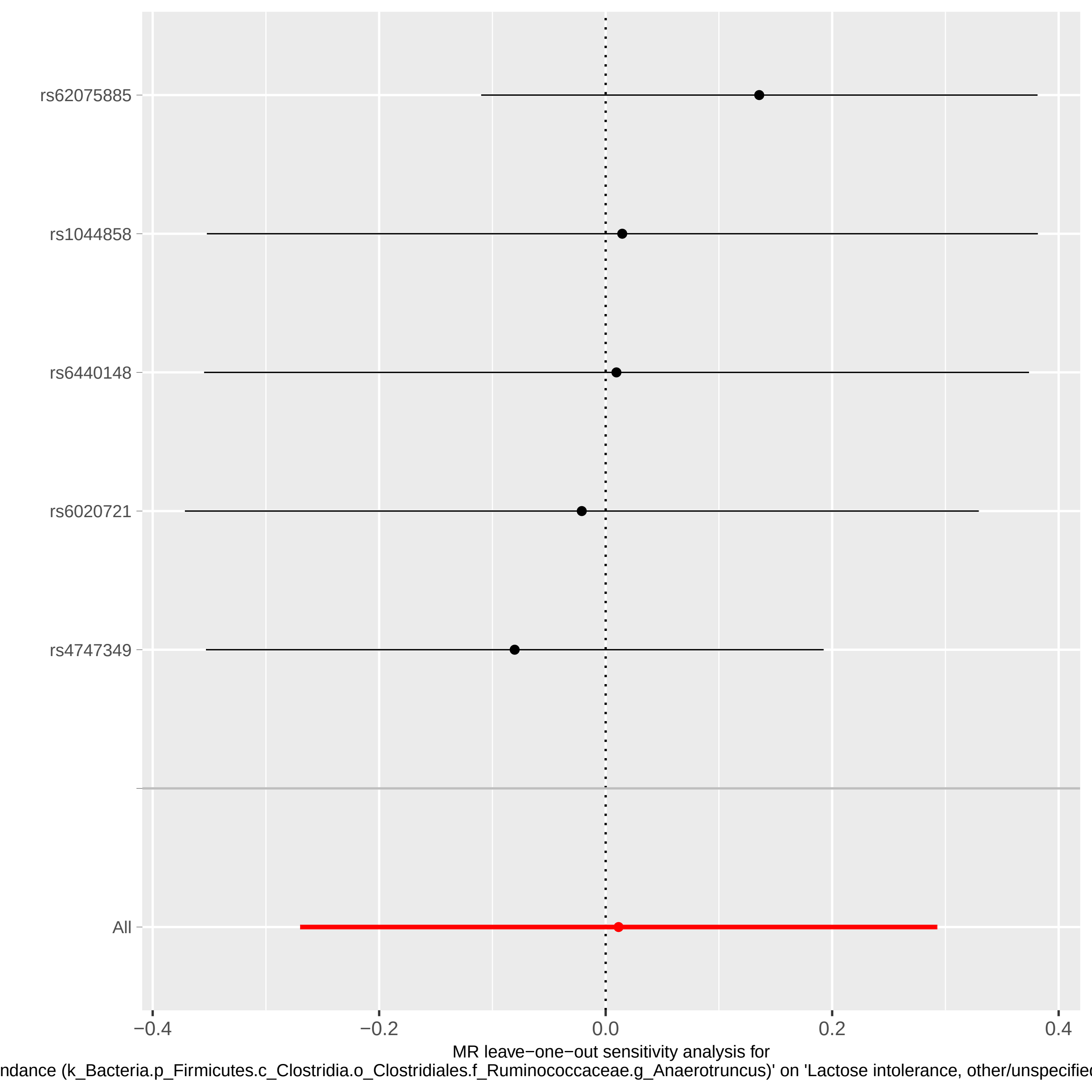
rs62075885
rs1044858
rs6440148
rs6020721
rs4747349
All
−0.4
−0.2
0.0
0.2
0.4
MR leave−one−out sensitivity analysis for
ndance (k_Bacteria.p_Firmicutes.c_Clostridia.o_Clostridiales.f_Ruminococcaceae.g_Anaerotruncus)' on 'Lactose intolerance, other/unspecifie
